# Participant experiences with a text message and contingency management intervention for alcohol use during pregnancy and lactation in Cape Town, South Africa

**DOI:** 10.21203/rs.3.rs-4900516/v1

**Published:** 2024-09-12

**Authors:** Lesley-Ann Erasmus-Claassen, Noluthando Mpisane, Petal Petersen Williams, Felicia A. Browne, Bronwyn Myers, Wendee M. Wechsberg, Charles David Heber Parry, Shantae N. Taylor, Yukiko Washio

**Affiliations:** South African Medical Research Council; South African Medical Research Council; South African Medical Research Council; RTI International; South African Medical Research Council; RTI International; South African Medical Research Council; RTI International; RTI International

**Keywords:** prenatal alcohol use, Fetal Alcohol Spectrum Disorders, contingency management, text messaging, maternal health

## Abstract

**Background:**

The Western Cape region of South Africa has one of the highest global rates of Fetal Alcohol Spectrum Disorder (FASD), underscoring the urgent need for effective interventions. This qualitative study explores pregnant and lactating participants’ perceptions and experiences of a text message and contingency management (CM) intervention.

**Methods:**

The study involved post-intervention interviews with 10 pregnant participants and 10 post-partum lactating participants. Coding and a thematic analysis approach were applied to the collected data using NVivo 12.

**Results:**

Four main themes emerged from the analysis: (1) program experiences; (2) intervention components; (3) health behavior change; and (4) recommendations for program improvements. The participants valued financial incentives and health-promoting text messages, noting reduced alcohol consumption.

**Conclusion:**

The findings highlighted the potential benefits of the intervention in improving individuals’ health behaviors. However, logistical barriers and the need for expanded support services were identified, emphasizing the importance of refining intervention strategies in resource-limited settings.

## Background

Alcohol consumption during pregnancy remains a public health concern in South Africa, with a national prevalence rate of maternal alcohol consumption at 3.7%(^1)^. Studies in the Western Cape estimate alcohol consumption rates ranging between 20.2%^(2)^ and 37%^(3,4)^ among women receiving antenatal care. Prenatal alcohol use is associated with a multitude of adverse effects during pregnancy and birth outcomes including miscarriage, stillbirth, preterm or premature birth, lower infant growth outcomes relating to weight-, height- and head circumference-for-age^(5)^, intrauterine growth restriction, Fetal Alcohol Spectrum Disorder (FASD) and sudden infant death syndrome^(6–9)^. FASD refers to a spectrum of neurodevelopmental disorders caused by in-utero alcohol exposure^(10)^. The reported prevalence of FASD in South Africa is among the highest globally, ranging between 29 to 290 per 1,000 live births^(11)^, with the Western Cape region reporting a prevalence ranging from approximately 13.6 to 20.9% (135.1–207.5 per 1000)^(12,13)^.

Heavy episodic drinking is not restricted to individuals who are pregnant^(1,14,15)^ it has also been reported among individuals who are breastfeeding^(16–18)^, with 71% of individuals in one study reporting alcohol consumption during the postpartum period^(19)^. Alcohol use while breastfeeding can negatively affect breastfeeding duration, lactational performance, breast milk production, and infant neurocognitive development^(16,20)^. Studies have shown that post-partum drinking and breastfeeding can lead to lower birth weight, lower verbal IQ scores and more anomalies compared to children whose mothers abstained from alcohol while breastfeeding^(19)^.

Various factors are associated with heavy drinking during pregnancy including poverty, unemployment, and food insecurity^(15,21)^; maternal age; partner’s level of education; unwanted and unintended pregnancy; a pre-existing alcohol use disorder; HIV status^(22)^; intimate partner violence^(5)^; lack of support from family^(15,21)^; and societal pressure^(18,23–25)^. Despite education efforts, individuals persist in drinking during pregnancy, highlighting the urgent need for targeted interventions. Comprehensive strategies, that integrate education, health promotion, and evidence-based approaches, are crucial for fostering and supporting positive changes in alcohol-related behaviors^(26,27)^. Contingency management (CM) is regarded as one of these evidence-based approaches and is used to address alcohol and other substance use-related behaviors by rewarding individuals with incentives such as money, prizes, or vouchers, for positive changes in their health behavior^(28)^. In the context of substance misuse, people sometimes receive reinforcers in the form of money for testing negative for alcohol on their urine test^(29,30)^. In addition to CM, text messaging and other mobile health (mHealth) initiatives have been used to improve appointment attendance^(31)^ improve adherence to treatment regimens, educate patients and promote behavior change^(32)^. Despite these studies, extensive work still needs to be done to prevent alcohol consumption during pregnancy and lactation, particularly in the Western Cape Province. This study aimed to explore participants’ perceptions of and experiences with a text-messaging and CM intervention with individuals reporting hazardous alcohol use during pregnancy and lactation.

## Methods

We conducted a qualitative study nested within the Maternal Alcohol Reduction Intervention in South Africa (MARISA) study to understand participants’ perceptions and experiences of a text messaging and CM intervention. The MARISA study, including the development and pilot test of this intervention, is fully described elsewhere^(33)^. This qualitative study is presented in line with the COnsolidated criteria for the REporting Qualitative research (COREQ) guidance^(35)^.

### 1. Participant recruitment

Twenty out of a sample of 60 participants, 10 who were pregnant and 10 who were post-partum and breastfeeding at the time of main study enrollment were randomly selected from the main sample based on a computer-generated random selection of numbers, to be interviewed after completing participation within the main study.

All the selected participants were enrolled in the main study and received the pilot intervention. From the random selection, two women who were selected had to be replaced because one withdrew from the main study and the other could not be found during tracking efforts. Both of these selected participants were replaced through another random selection. All of these potential participants consented to a one-hour interview during their original study participation. Interviews were conducted in a private room at the two identified healthcare facilities or in the participant’s home. The interviews were completed with only the participants and the interviewer present.

The interview guide questions were aimed at exploring the participants’ general experience of being a participant in the study, their perceived behavioral adaptations (if any), and their views on the intervention components i.e. CM through financial incentives and receiving weekly health-promoting text messages in short messaging service (SMS) format. Participants were asked to share their perceptions of the content of the text messages and whether the messages impacted their general health behaviors. Participants’ satisfaction with the intervention, its impact, areas of improvement, and recommendations relating to improving future intervention programs were also examined.

### 2. Procedures

Interviews were conducted by the first author (LEC), a native of the Western Cape, who has a post-graduate degree in psychology, is bilingual and has extensive qualitative research experience. At the time of data collection, she fulfilled the role of Project Coordinator on the MaRISA study and had no prior relationship with any of the participants. The participants were asked to provide written informed consent prior to commencing the interviews. Interviews were conducted in person from October 2022 until March 2023. Interviews lasted up to 30 minutes and were audio recorded and translated while being transcribed. Since the interviews were conducted in both English and Afrikaans, audio recordings of the interviews in Afrikaans were transcribed and concurrently translated into English.

The established semi-structured interview guide was developed to focus discussion points during the post-intervention key informant interviews, but participants’ responses determined further exploration and other discussion points. Participants were reimbursed for their time at ZAR150 (~ USD7.95 [ZAR18.86/USD1]) per participant.

### 3. Data analysis

Reflexive thematic analysis (TA) was used to analyze the data^(36,37)^ in the format of English transcriptions. According to Braun and Clarke^(38)^, coding reliability approaches prioritize early theme development and consider coding as identifying evidence for predetermined themes, whereas reflexive approaches such as TA, involve later theme development from codes, emphasizing shared meaning emerging from the researcher’s interpretative effort. Themes are constructed rather than inherent, and the coding process is organic and subjective, requiring critical reflection from the researcher. In this context a hybrid approach to coding was used, with a deductive approach used to code key concepts asked about in the interview guide and an inductive approach that allowed new codes to emerge from the data. The approach involved six recursive phases : (1) data familiarization and taking notes;(2) data coding; (3) generating initial themes from codes and collated data; (4) reviewing and developing themes; refining, defining and naming themes; and, (6) writing the results in the final phase^(36)^.

NVivo 2020 software was utilized to store and manage the data as well as facilitate coding. This approach also included recording memos, throughout the data collection and analysis process to record thoughts, notes, and observations. The first author (LEC) conducted the initial process of familiarization by reviewing the transcripts. Both the first author and the second author, NM, discussed the initial code definitions and coded the first two transcripts independently. Hereafter, the authors reconvened to discuss the refinement of the themes and the emergent sub-themes, as well as sharing notes reflecting on their personal positionality and understanding of definitions. Combining codes into overarching themes and redefining themes also formed part of this process. A Cohen’s Kappa coefficient score of 0.89 provided a measure of inter-coder agreement/reliability. Independent coding then resumed for all the transcripts. Any coding disagreements were resolved through discussion and consultation with one of the authors (PPW).

## Results

Participants ranged in age from 19 to 43 years, with a mean age of 28 (SD = 2.12); fourteen (70%) women self-identified as Colored (of mixed-race ancestry), and 6 (30%) women self-identified as Black African. Approximately 95% of the sample reported 12 years or less of education and 65% of participants were unemployed at the time of the interview. The majority of participants (75%) indicated that they had a main relationship partner at the time of the interview.

Four themes were generated from the interview data: (1) program experiences; (2) intervention components; (3) health behavior change; and (4) recommendations for program improvements. Program experiences encompassed all relevant descriptions of the participant’s experience being part of the program, their likes and dislikes, the lessons they have learned, as well as their perception of the overall intervention. The intervention components’ theme focused on participants’ views of the alcohol use monitoring process, perceptions of contingency management and the impact of the health-promoting text-messages. Health behavior change provides an overview of the adaptations that participants could make and the theme of recommendations for program improvements describes the key elements participants felt can be added to develop a well-rounded program.

### 1. Program experiences and perceptions of the intervention

The intervention program was not easy for all the participants to commit to and follow because of recent traumatic experiences, geographical access to the healthcare facilities and other similar barriers. For one of the participants, it was particularly difficult to follow the intervention program since she had lost her home due to fire amid participation, but during the post-intervention interview, she expressed that she still had a positive experience due to the tenacity of the field staff in their tracking efforts to find her: ‘During that time, I felt very different, and that time was the most difficult for me, that the place burnt down. And I was with my aunt for a short time. But they kept on looking until they found me. They left messages and so, but they took the trouble to reach me.’ (Tracey, 38 years old)

Some participants, especially pregnant participants, expressed that they did not like traveling to the health care facilities for their twice weekly alcohol monitoring (intervention) appointments since the activity tired them out: ‘It was the travelling … We live far so we had to travel and come to the clinic almost two times in a week so I would get tired quickly. That is the one thing that made me get tired of having to keep coming to the program.’ (Lizelle, 25 years old)

However, the travel assistance offered to address geographical barriers was also perceived as an additional incentive by some participants: ‘…that transport money also helped a lot. That ten rands helped a lot…and actually that was really great, because if it was winter, here, the taxi would be just right here.’ (Janice, 36 years old)

Despite their awareness of the risks associated with drinking during pregnancy, a significant portion of participants admitted to consuming alcohol. They acknowledged receiving information from health education materials and healthcare providers at the facility where they booked for delivery. Many of these participants emphasized the value of the intervention, noting its role in reducing or completely halting their alcohol consumption. ‘What I learned is just that it is not necessary to use alcohol during your pregnancy, it would be better if you do without it than to have it, you will have to bear the side effects. The poor little one will suffer at the end of the day. You will end up having an alcohol syndrome baby.’ (Melokuhle, 29 years old)

In addition, participants who were post-partum and breastfeeding reported gaining new insights into the potential impact of alcohol use while breastfeeding, like Emma, 23 years old, who shared, ‘When you breastfeed, when you drink alcohol and breastfeeding, you are giving the baby also the alcohol, so …it’s not healthy for the baby.’. Whereas another participant shared how her perception had evolved while participating in the program. ‘I knew now that it was dangerous to drink when you breastfeed, not that I did not know it, but it gave me a bit more insight.’ (Janice, 36 years old)

The participants shared how participation in the intervention program taught them to make changes to their drinking behaviors. One participant reduced her alcohol consumption due to the intervention, but her reduction also had a positive impact on her social network with one friend also reducing her alcohol consumption: ‘My one friend doesn’t drink anymore, even though her child is big … She says it is from you not wanting and buying beer all the time. Because I used to say to her, ‘E’ come let’s buy a box. Yes, I just used to buy a box. Then the two of us combine, then she says come we buy a box. Then we buy a box (referring to boxed wine) … But now…she also stopped … Now, no I don’t want wine. I don’t drink anymore.’ (Nontsikelelo, 27 years old)

### 2. Intervention components: Alcohol use monitoring and abstinence

The intervention included three key components: (1) alcohol use monitoring and abstinence, (2) contingency management (CM), and (3) health promoting text messages. With regards to alcohol use monitoring and abstinence, the participants shared varied experiences attending alcohol monitoring appointments at healthcare facilities but mainly acknowledged the positive impact of monitoring on reducing alcohol consumption. Financial incentives under CM were highly valued and health promoting text-messages, delivered through short messaging services (SMSs), served as a motivational tool for behavior change, instilling a sense of pride and purpose. Participants were sent weekly text messages during the program, which included generic health-promoting messages.

A small number of participants reported that they struggled to reduce their alcohol consumption and that the testing was a disincentive to attend the healthcare facility as they were concerned that their alcohol use would be detected. As shared by Grace, 43 years old: ‘To attend the program by using alcohol was very difficult for me, to be honest, it was very difficult, sometimes I didn’t want to come here …. Yes, it was tough … a little tough, yes … I am working as well … very difficult for me, yes.’ (Grace, 43 years old)

### 3. Intervention components: Contingency Management (CM)

Several participants reported that regular alcohol monitoring helped them to reduce their alcohol consumption because they knew they were going to be tested for alcohol use, such as Casey, 19 years old, who found it manageable and credited it with reducing their alcohol consumption. ‘It wasn’t difficult, … because fortunately I don’t live too far, I live near to the clinic, so it wasn’t difficult for me. It actually helped me a lot because I use to be a strong drinker and I came down from it quite a lot and it made me not drink, when I knew I had to be there at that time, because I knew I had to come and give my urine.’ (Casey, 19 years old)

Participants appreciated the financial incentives of the CM component and it served to motivate positive behavior change. For instance, Kayden used the incentive to support her children while unemployed.: ‘Like when I got that money … I could go and take out the lay-by. And then my other two kids, I bought each one of them something. So, it helped me a lot because I’m not working at the moment … [was] a big help for me yes.’ (Kayden, 33yrs old)

Participants shared that receiving financial rewards for abstaining from alcohol use helped them to stay motivated. ‘Because it was motivating us a lot to stop drinking alcohol and get some money so that we can buy things that we need to buy for ourselves during the pregnancy’. (Lizelle, 25 years old)

### 4. Intervention components: Health promoting text messages

Some of the participants perceived the content of the text messages to be helpful and expanded their knowledge base: ‘… it made me realize that the wine goes into the breastmilk and at the end of the day, the baby will drink from my breast. So, yes it did make me think a lot.’ (Casey, 19 years old)

Iminathi, 23 years old, shared that the text messages made her feel proud of herself and further encouraged her to motivate others in a similar situation to join the program.: ‘Yes, I received SMSs from you … these SMSs were making me to be proud of me, hey, and to know what I have to do for my life. Yeah, from these SMSs they make me to tell others that they are supposed to join in the group if they got a problem like mine, they have to join so they can be alright’ (Iminathi, 23 years old)

Notably, most of the participants reported barriers to receiving these text messages. These barriers included not having access to a mobile phone and frequent change in phone numbers.

### 5. Health behavior change

The participants reported that the MARISA intervention helped them make changes to their alcohol and tobacco use, and to their general health, habits and behavior. Some of the participants expressed that joining the program helped them quit alcohol use during pregnancy and while breastfeeding. For example, Nontsikelelo admitted to heavy drinking during her pregnancy, however, once she entered the program, she was able to abstain from alcohol and permanently quit smoking.

Participants described how these changes in their alcohol use helped improve their living conditions and their relationships with their families. Participants described that during enrolment of the program, they did not drink on weekends and were able to spend more time with their families. Some participants thought that these changes contributed to a healthier pregnancy. Kayden, 33 years old, stated that she had ‘left the alcohol at the end of the day, and I have a healthy baby today.’ Grace, 43 years old, shared a similar sentiment that attending the program was in her best interest. It helped her to drink less, especially now that she has obtained full-time employment: ‘At the end of the day, no I tried, when I got deeper into the program, then I am getting a sense of it’s for my best, uhm so I have to attend. So, for me, it’s known I am drinking less and especially now I am working full-time. So, I am drinking less and weekends, I am going to work so there’s no time.’ (Grace, 43 years old)

### 6. Recommendations for program improvement

Participants expressed their interest in alternative types of rewards such as vouchers from local supermarkets or retailers. As Carmine (22 years old) stated: ‘you can sometimes add vouchers, hey. And things like that, maybe for the babies, vouchers, for the things when the person is pregnant, they get cravings right, maybe a voucher or something, yeah.’

Other suggestions to improve the intervention program included education on family planning to prevent unplanned and/or unwanted pregnancies and testing for other substances in the program since women were only monitored for alcohol use with an ethyl glucuronide (EtG) test: ‘You can educate on substances like drugs and so … not only alcohol, but preventing pregnancy …’ (Carmine, 22 years old) and ‘For me maybe, a drug testing … I would like a drug test to be added, then it would be better for me.’ (Nontsikelelo, 27 years old)

Expanding the intervention components to include behavior change counseling and incorporating recreational activities to address boredom were some of the other suggestions that participants had to improve the received program. ‘You need to sit with them and counsel them. To show them how it destroys them, help them … And do activities … Just to keep, uhm the people busy …’ (Emma, 23 years old), and ‘They need counselling. You can put more information about … that drugs are not right for your health and then you can use, you can actually put more information in and try to help them … To not walk around … not think about that, that I have to smoke now.’ (Abigail, 21 years old)

The need for support groups incorporated into intervention programs was raised by the majority of participants, even though participants were demographically from different communities within the Western Cape: ‘Here are no support groups, nothing, there’s nothing. Look here in [my community], there’s no life, there isn’t even any work, no income. It is needed because here is nothing.’ (Likhona, 36 years old)

Future intervention programs that include main partners should focus on strategies to prevent intimate partner violence (IPV), teach men better-coping mechanisms other than using violence and focus on educating on gender roles, as one participant elaborates about sharing responsibilities within a relationship: ‘With my partner then there must be a program for the abusive partner. To maybe say the man must do that, is all that the woman wants. You can’t expect from your side alone and then you don’t do your part … [speak about] Gender roles … One hand helps the other hand…’ (Tracey, 38 years old)

Similarly, support groups can explore the positive characteristics of being a supportive partner: ‘Maybe not to abuse your woman. Or sleep around. Cheating and such stuff. Or to be there for your child. Even if you’re not with the mother but support your child. Because some fathers, they want to be in the child’s life. But then they get avoid [ignored] by the mum or she’s getting avoided [ignored] by the other mother (grandmother). And then you get that father that just don’t care.’ (Sarah, 23 years old)

## Discussion

The purpose of this study was to explore the perceptions of participants who received the MaRISA intervention. Most participants reported positive outcomes, including reduced alcohol consumption, improved overall well-being, and positive impacts on their peer network. Notably, some women reported sustained abstinence from alcohol post-intervention, highlighting its potential for long-term benefits. Additionally, supporting literature from various studies affirms the Contingency Management approach’s effectiveness in curbing excessive alcohol consumption across different demographics^(28,29,39)^. However, some participants cited logistical difficulties, such as transportation issues and travel distance to the healthcare facilities, which made the bi-weekly urine sample submissions for the program cumbersome.

Contingent financial rewards from the MaRISA intervention for abstaining from alcohol supported participants in meeting family needs and starting business ventures, leading to health improvements, behavior changes, and long-term financial security. The success of such monetary and suggested non-monetary incentives in this and other studies^(40,41)^ underlines the effectiveness in encouraging sustained recovery and healthier choices during key life stages^(42,43)^.

Participants appreciated receiving weekly texts about the risks of drinking alcohol during pregnancy and while breastfeeding, finding the messages educational and motivational. Similarly, a study conducted by Lau and colleagues^(44)^, among pregnant women attending a primary health care facility in Cape Town, revealed that text messages containing antenatal health information served as a source of positive reinforcement for the participants and helped to improve health-related behaviors. Although participants who received the health-promoting themed text-messages weekly found it useful and motivating, it is important to note that not all the participants received the text messages. Some, however, were missed due to a lack of mobile access or changing phone numbers frequently. Mekonnen et al.^(45)^ speak to this, having found that low mobile ownership is a barrier that could have an impact on mobile health intervention implementation. Addressing these barriers are crucial for the success of such digital interventions in resource-limited and low socioeconomic settings^(46,47)^. Despite these challenges, overall findings indicate that text messaging shows promise in promoting healthier behaviors among pregnant and lactating individuals.

To enhance the program, participants suggested adding counseling for behavioral change, education on family planning^(48)^, testing for other substances, and incorporating other recreational activities^(43,49)^. There was a call for a focus on intimate partner violence and gender roles, linking alcohol use to IPV^(50)^ and substance use as a way to cope with abuse^(22)^. Furthermore, individuals are most likely to drink during pregnancy if their partners are not good sources of support^(50)^. These user recommendations are backed by research^(4,15)^, which emphasizes the importance and effectiveness of health education^(4,15,48,49,51–53)^, as well as the impact that incentive-based interventions for reducing alcohol use can have during pregnancy and lactation^(29,30,54,55)^.

It is important to consider the limitations of this study when interpreting its results. This sub-study focused on exploring the perceptions and experiences of 20 participants from only two communities in the Cape Metropole of the Western Cape Province of South Africa. Therefore, the representativeness of findings for individuals who consume alcohol while pregnant or post-partum and who are breastfeeding in South Africa remains limited.

## Conclusion

This sub-study provides valuable insights into how a text-messaging and CM program could aid South African pregnant and postpartum individuals in reducing alcohol use, also positively affecting their peers. Suggestions for program enhancement included integrating behavior change counseling, recreational activities and using a support group format. The findings underscore the value of participant feedback in refining interventions for alcohol use reduction among pregnant and breastfeeding individuals.
